# Selected Errors in Spatial Measurements of Surface Asperities

**DOI:** 10.3390/ma17122918

**Published:** 2024-06-14

**Authors:** Karol Grochalski, Dominika Podbereska, Michał Wieczorowski, Rafał Talar, Wiesław Graboń

**Affiliations:** 1 Faculty of Mechanical Engineering, Poznan University of Technology, ul. Piotrowo 3, 60-965 Poznan, Poland; dominika.podbereska@put.poznan.pl (D.P.); michal.wieczorowski@put.poznan.pl (M.W.); rafal.talar@put.poznan.pl (R.T.); 2 Faculty of Mechanical Engineering and Aeronautics, Rzeszow University of Technology, 35-959 Rzeszow, Poland; wgrabon@prz.edu.pl

**Keywords:** surface asperities, thermal errors, elongation, interferometric measurement, interior thermal influences, center of gravity

## Abstract

This work presents issues related to selected errors accompanying spatial measurements of surface roughness using contact profilometry. The influence of internal heat sources, such as engines or control electronics, on the thermal expansion of the drive responsible for the measurement probe’s movement in the X-axis direction was investigated. In terms of starting measurements on a thermally unstable device, the synchronization error of individual profile paths was 16.1 µm. Based on thermographic studies, the time required for full thermal stabilization of this drive was determined to be 6–12 h from when the device was turned on. It was demonstrated that thermal stabilization of the profilometer significantly reduced positioning errors of the measurement probe on the X-axis. Thermal stabilization time should be determined individually for a specific device variant. This research also determined how changes in the center of gravity caused by the measurement probe’s movement affected the overall rigidity of the profilometer structure and the leveling of the tested surface. Laser interferometry was used for this purpose. The determined vulnerability of the profilometer structure was 0.8 µm for a measurement section of 25 mm. Understanding the described relationships will reduce errors associated with conducting measurements and preparing equipment for tests. Additionally, it will enable the correct evaluation of surface geometry.

## 1. Introduction

Evaluating surface texture is essential to reliably characterize product quality, functional properties, and tribological characteristics [1]. The accuracy of surface topography measurements and the analysis of collected data are crucial to assessing the mechanical properties of industrial surfaces [2]. They often constitute an integral part of the process control [3]. There are many methods for measuring surface texture, which can be divided into two main categories: contact and non-contact [4,5,6,7,8,9]. Contact methods, with the most common devices being stylus profilometers, use a diamond measurement tip that moves across a surface and records vertical deviations. Although these are well-known methods, they are sensitive to vibrations, noise, and other disturbances [8,10,11] as well as thermal effects [12,13,14,15] and geometric errors in the measurement tip [16,17]. Compared to other methods, they are time-consuming [18], have low measurement throughput, and require direct contact with the sample, which can cause scratching and damage to the surface being examined [19,20]. To overcome the limitations of contact methods, non-contact methods for measuring surface texture have been developed, mainly based on optical systems. These techniques use a beam of light reflected from the surface under examination to analyze irregularities. Optical methods include chromatic confocal microscopy, focus variation instruments, white light interferometers, atomic force microscopy, and several others [4,6,21,22,23]. Errors in surface topography measurements can be caused by the measured objects, measuring equipment, the environment, software, and measurement methods [24], which affect various surface textures [21].

Although optical methods for measuring surface roughness offer several advantages, such as short data acquisition time and high accuracy, their results may also be subject to errors. These errors can have both external and internal characteristics [25,26]. Optical measurements of surface texture rely on the use of light, its intensity, or the direction of incidence, which determine the accuracy of the obtained results. Too low a light intensity may prevent the detection of all surface points, resulting in their replacement with smooth shapes. Missing data are usually interpolated based on adjacent points. Conversely, too high light intensity can distort the results (causing an increase in measured parameter values) [27]. The direction of illumination on the surface under examination also directly affects parameter values in optical profilometry [25]. Changing the angle of incidence can cause changes in height, hybrid, and functional parameter values. The selection of surface illumination parameters may be hindered by light reflections from the measured surface. Sharp edges on the surface under examination generate artifacts in the form of high and narrow “peaks” that do not reflect the actual surface topography [7,28,29]. This problem can be addressed using a polarizer in the optical system or by changing the intensity of the sample’s light incidence. The orientation of the polarizer relative to the sample with a directional surface texture significantly affects measurement results. Different angular settings of the polarizer axis generate different surface parameters and distort the cross-sectional profile, making it difficult to accurately reproduce the pattern’s topography [25]. Another issue is the presence of unmeasurable points. According to Pawlus et al. [27], these points distort the values of surface texture parameters, affecting the representation of surface texture parameters and leading to a false assessment of the quality of processed elements.

Due to its widespread usage, stylus profilometry has been the focus of many studies. Despite its numerous advantages, this technique is susceptible to various disrupting factors, particularly environmental influences, such as temperature changes, for example, from conventional binary air conditioning systems.

Adamczak et al. [12] studied the impact of temperature variations over time on surface stereometry measurements. They tested the effects of a profilometer cover designed to minimize temperature fluctuations. The cover reduced the temperature gradient, thereby decreasing interference, but did not entirely eliminate it. Cyclical temperature changes during measurements affect the waviness parameters along the Y-axis. Ambient temperature changes also influence the elongation of both the tested object and the measuring device during surface topography measurements. This effect is negligible in short, two-dimensional profile measurements but causes errors in spatial measurements lasting several hours. Grochalski et al. [13] also conducted studies in a specially designed climate chamber to examine the impact of ambient temperature changes on surface topography measurement errors. Their study found that ambient temperature changes significantly affect the accuracy of surface topography measurements, with even a 1 °C change leading to noticeable errors in amplitude parameters. Unstable conditions can affect both the measured element and the profilometer’s structure. In another study, Grochalski et al. [14] also demonstrated that internal heat sources, originating from the contact profilometer drives, affect measurement accuracy. However, the application of thermal stabilization can reduce these errors by about 90%. The conducted studies [15] showed a correlation between temperature changes and measurement results of measured surfaces. Ambient temperature variations significantly affect surface measurement results and measurement accuracy, thus impacting the reliability of the obtained results. Temperature fluctuations can cause geometric and shape errors, highlighting the importance of controlling and stabilizing temperature conditions during surface topography measurements [30]. Path synchronization errors are also a significant issue. The authors of the same study addressed this topic. Wieczorowski et al. [30] noted that these errors cause significant changes in peak location expectations and distort the functional character of the measured surface, affecting the fidelity and accuracy of the surface topography. One solution is to stabilize the profilometer’s operating conditions (for several hours) before starting measurements and appropriately adjust the data acquisition speed.

This issue was also discussed in a publication [16] by Yin et al. They used a system consisting of a precision scanning stage, a measurement system, a microstylus, and a vibration isolation table to conduct studies on samples with triangular microstructures. The system accurately measured the shape, depth, and period of the microstructures, correcting for errors resulting from the sample’s tilt and the measurement stylus tip radius. These measures demonstrated the feasibility of the proposed measurement system for microstructures with complex surface topographies. A different approach to this issue was proposed by Lee and Cho [17], who suggested that the radius of the profilometer’s measuring tip distorts measurements, necessitating selection criteria based on the sample’s surface texture characteristics to improve results reliability. This hypothesis was confirmed through simulations and comparisons with a roughness tester. Based on the analysis of the results, the effective frequency range components of the profilometer were determined. Criteria for selecting the measuring stylus tip radius were also proposed considering the sample’s surface texture characteristics.

Additionally, typical errors of measuring styluses can be predicted by mechanical filtering of the measuring stylus tip or flight of the measuring stylus [5]. High-frequency noise in surface topography measurement results can disrupt surface texture parameter values, leading to incorrect interpretations. Therefore, appropriate selection of the filtering method is important depending on the type of analyzed surface texture [11].

Another important aspect is the speed of the measuring stylus tip, which affects surface roughness measurement results [31]. Higher speeds can lead to increased dynamic effects and vibrations, impacting the accuracy of the obtained measurement results. A larger tip radius smoothes the measured profile, reducing the influence of small surface irregularities. If the speed is too high, the stylus tip may lose contact with the measured profile, a phenomenon known as “flying”, leading to an incorrect representation of the profile.

To understand the precise interactions of thermal mechanisms on profile measurements, Grochalski et al. proposed a comprehensive thermal diagnostic of profilometric measurement instruments [13]. They proposed a thermographic method to assess technical condition and/or map the thermal fields of examined objects. This approach provides the user with information about areas that may affect the thermal susceptibility of the profilometer’s construction and, based on this, infer potential errors in representing surface asperities.

A review of the relevant literature indicates that many recent studies do not identify the causes of errors in contact or optical measurements related to surface geometry. These works rely on already known sources of errors, which are examined in specific, limited cases. Most studies focus on quantifying and correcting various sources of errors using broadly understood filtration methods [32,33] or AI [34]. This research gap motivated the authors of this study to conduct detailed research on the sources of these errors.

Considering that contact measurements are well-known and widely used in industrial applications, this study focused on thoroughly understanding the mechanisms behind thermal interactions affecting the accurate representation of geometric surface textures and the kinematics of motion influencing the susceptibility of profilometer construction. The main goal of this study was to determine how the thermal expansion of the profilometer’s X-axis drive, along with dependent elements responsible for the movement of the measuring tip, affects the spatial representation of the surface. Additionally, the influence of disturbances from profilometer construction’s susceptibility to changes in the center of gravity position during movements of the X-arm with the measuring tip was examined. This study builds upon and complements research related to errors in contact profilometry [13,14]. Dependencies resulting from thermal expansion directly affect the parametric values of surface geometry texture. Understanding the nature of disturbances will help eliminate or significantly reduce widely interpreted measurement errors, leading to a more accurate representation of surface texture.

## 2. Purpose of Research, Methodology, Measuring Track, and Devices

As mentioned in the introduction, the development of manufacturing processes for machine and equipment components is closely linked to improving inspection methods and determining functional surface characteristics. This research was conducted when the device was turned on and thermally unstable. Such measurements helped determine the influence of internal thermal conditions on surface representation accuracy.

The research methodology is twofold:

– The effect of thermal expansion of contact profilometer elements on arm displacements responsible for movement along the X-axis of the measuring head from when the device was turned on;
– The profilometer construction’s susceptibility to changes in the center of gravity position of the X-axis drive caused by the movement of the measuring tip.

**Figure 1:**
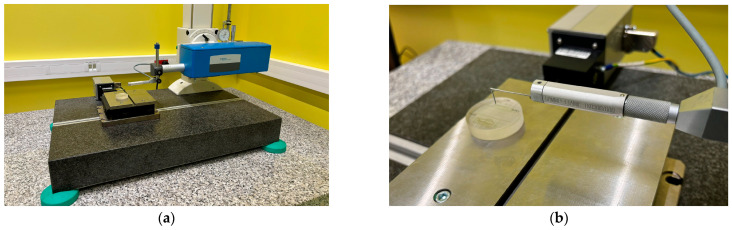
Measurement setup: (**a**) Hommel T8000; (**b**) test object—reference glass plate.

Surface texture analysis was performed using the contact method, with a Hommel T8000 device equipped with a TKU 300 measuring head (Jena, Germany) and a measuring tip with a diamond cone angle of 90 degrees (Figure 1).

The measured element was a reference glass plate PEN-10-1 6820101 (Germany) with a reference crack width of 0.125 mm, depth of 7.6 µm, and flatness defined by a λ/20 parameter.

The temperature of profilometer components was measured by contact temperature sensors placed at heat source (control electronics, drives) locations. Semiconductor sensors DS18B20+ (Dallas, TX, USA) were used to monitor both ambient temperature and temperature inside profilometer components. The temperature sensor resolution was set to 12 bits, corresponding to increments of 0.0625 °C, respectively. Before measuring the profilometer and its surroundings, calibration of the used sensors was also conducted using an MLW U8 ultrathermostat (Germany). Temperature ranges of the calibrated ultrathermostat were set for values occurring inside the profilometer structure between 20 °C and 35 °C. Calibration was also verified using an independent certified temperature sensor TESTO 925 (Titisee-Neustadt, Germany).

The distribution of thermal fields was examined using a thermal imaging camera, recording thermal changes on the profilometer surface. A FLIR T620 thermal imaging camera (Wilsonville, OR, USA) with a spatial resolution (in terms of instantaneous field of view) of 0.62 milliradians and a thermal resolution (in terms of temperature difference equivalent to noise) of 0.05 °C was used. The camera’s margin of error was ±2 °C or ±2% of the read temperature value (the greater of the two values is considered the margin of error) [35]. Additionally, to verify the accuracy of the camera’s readings and calibration, temperature references of perfectly black bodies LAND P550P and LAND P80P (Sheffield, UK) were used. The accuracy of the camera’s readings was measured after its full thermal stabilization as well as the thermal stabilization of the references. The set temperature values for the references were 20 °C and 35 °C, creating a range corresponding to the temperatures within the profilometer construction.

To measure the positioning of the measuring tip, as well as the thermal expansion of the X-axis movement arm’s kinematic system, a Lasertex (Inc.) (Wrocław, Poland) LSP30-3D laser interferometer with an external reference optical system, phase correction, 100 pm resolution, and a maximum measurement error under 0.5 µm/m was used. The interferometer determined the influence of internal heat sources, such as motors responsible for movement in individual axes and control electronics, on the thermal expansion of the profilometer in the direction of the examined axis. The interferometer system was thermally stabilized before data registration and the measurement path’s environmental conditions were constant.

### 2.1. Reproducibility of Positioning the Measuring Tip and Path Synchronization

**Figure 2:**
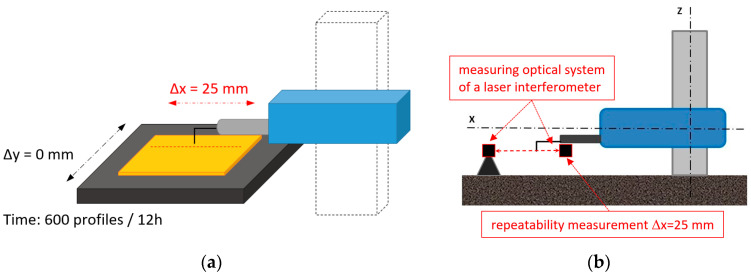
Schematic and measurement path for assessing the impact of thermal interactions on the profilometer: (**a**) methodology of measuring a single profile in the time domain; (**b**) measurement path using a laser interferometer.

To determine the influence of internal heat sources on the accuracy of path synchronization and positioning of the measuring tip, the same profile was measured 600 times on a flat parallel plate with a reference scratch (Figure 2a). For this purpose, the table movement responsible for motion along the Y-axis was disabled (Δy = 0). The positioning of the measuring tip was performed using a laser interferometer aligned with the X-axis of the profilometer arm. From the profiles recorded in this manner, the surface with the scratch was reconstructed, where each subsequent profile was represented in the time domain (Figure 2b).

**Figure 3:**
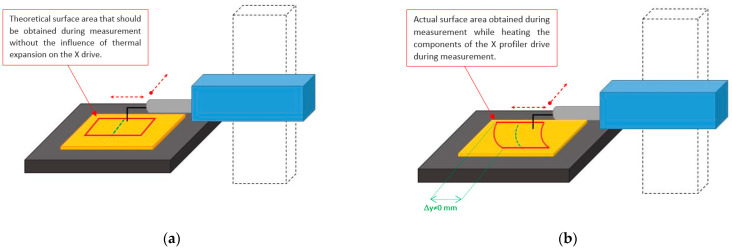
Diagram representing the results of the reference scratch: (**a**) reconstructed scratch under stable environmental conditions; (**b**) distorted scratch due to thermal effects and thermal expansion of the profilometer construction.

This type of study, in the case of undisturbed measurement conditions and the absence of thermal expansion effects, should theoretically obtain an undisturbed, straight scratch from the reconstructed surface over time (Figure 3a). In the event of a change in the profilometer arm length due to thermal expansion, the reconstructed scratch should have a non-linear character, as illustrated in Figure 3b.

In Figure 3, changes in the shape of the reference scratch depict increases in the temperature of the profilometer construction, causing the elongation of its components relative to the vertical column of the device. In such cases, the reproduced scratch will have a curvilinear character and be asymptotic until thermal stabilization of the device occurs. This relationship was experimentally confirmed.

### 2.2. Susceptibility of the Profilometer Construction to Changes in the Center of Gravity

Another significant factor influencing errors in contact surface measurements is the vulnerability of the profilometer’s construction to changes in the center of gravity. Changes in the center of gravity occur during the movement of the profilometer’s X-axis arm and the displacement of the measuring tip, head, and other elements during measurement. This type of disturbance directly affects surface leveling representation.

**Figure 4:**
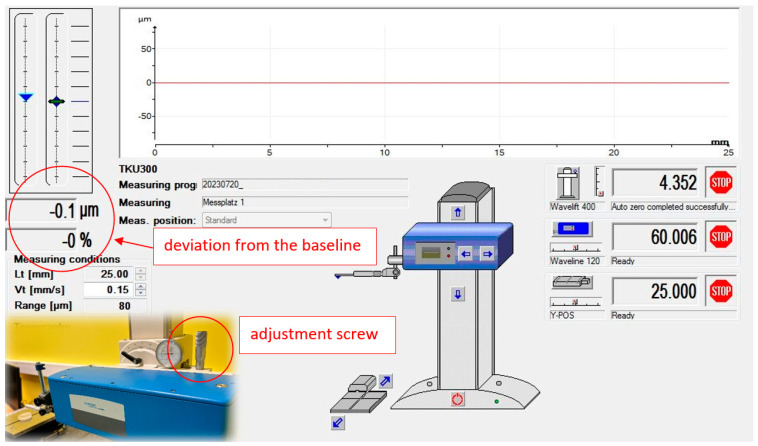
The TurboWave program window with the levelling indication of the drive unit highlighted.

The examination was conducted on a thermally stable profilometer, employing the same measurement methodology as described in Section 2.1. Measurements were obtained with the measuring head precisely leveled relative to the measured flat-parallel plate in a section without a reference groove. The leveling error of a 25 mm section did not exceed 0.1 μm, as inferred from the informational window of the TurboWAVE V7.59 program controlling the device (Figure 4). The measuring head was set according to the device’s global coordinates, with a starting point of 65 mm and an ending point of 40 mm, where the Hommel T8000 profilometer’s full range of movement in the X-axis direction was 120 mm. The measurement parameters were as follows: Z-axis (vertical) range ±80 μm, measured profile 25 mm × time (600 profiles/12 h).

**Figure 5:**
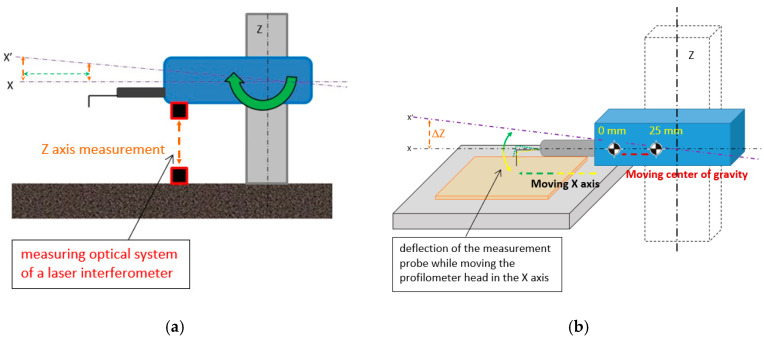
Method for determining the profilometer construction’s susceptibility: (**a**) measurement setup and methodology for determining the profilometer construction’s susceptibility; (**b**) representation of the profilometer construction’s susceptibility to changes in the center of gravity position.

The measurement of the construction’s susceptibility to deformation was conducted using a laser interferometer. One of its mirrors (reference) was placed on a granite plate, while the other (measurement) was on a rigid holder attached to the device’s body, as depicted in Figure 5a. The measurement mirror was positioned at a precisely defined location at a known distance from the X-axis drive. This axis serves as a precise leveling adjustment for the X-axis drive using a micrometer screw. The deviation of the measurement tip and the apparent deviation of the axis marked as X′, due to the movement of the profilometer arm, are schematically shown in Figure 5b.

Since the measuring tip remains in constant contact with the surface under examination, tilting the profilometer’s construction directly affects the representation of individual profiles and, consequently, the surface. The data are then depicted as unleveled. Consequently, the texture’s amplitude parameters may be erroneously represented. This potential for error is particularly significant for surfaces with low roughness parameters or step analysis.

Measurement data describing surface geometry as well as the analysis of the reference scratch were processed and analyzed using MountainsMap 10 software.

## 3. Results and Discussion

**Figure 6:**
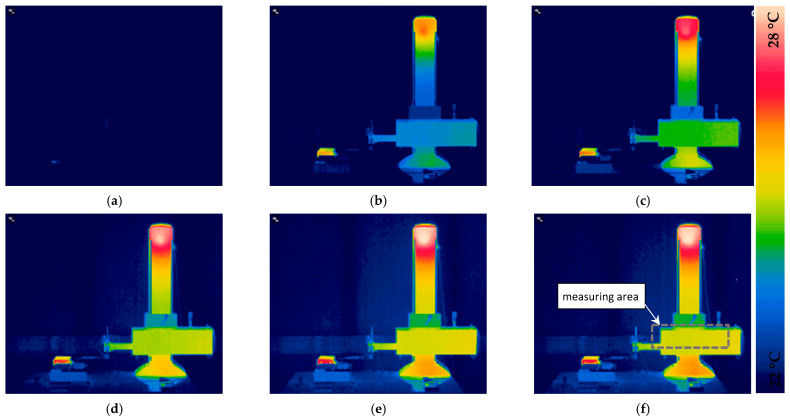
The heating of the profilometer construction due to internal heat sources when it is turned on. Thermal field distributions at (**a**) 0 h; (**b**) 2 h; (**c**) 4 h; (**d**) 6 h; (**e**) 8 h; (**f**) 12 h.

**Figure 7:**
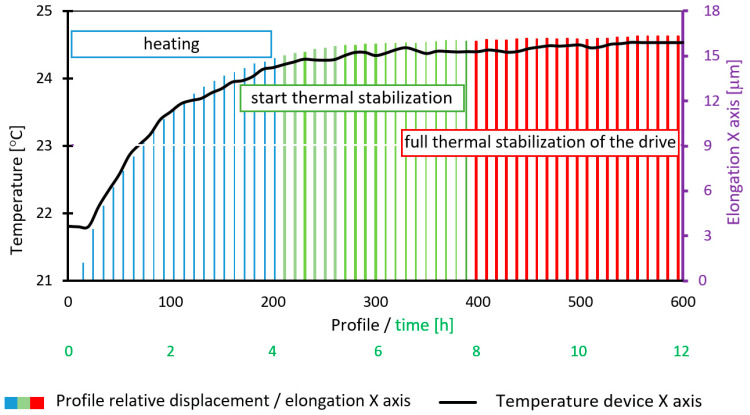
The relative displacement of the profilometer’s measuring tip as a function of temperature change, as measured by an interferometer.

The first stage of this study involved measuring the temperature of profilometer components, particularly the X-axis drive. Two independent methods were used for this purpose: a thermal imaging camera (Figure 6), whose readings are presented later in this paper (Figure 7). Thermograms also illustrate the distribution of thermal fields in profilometer components and heat flow over time (Figure 6). Supplementing the thermographic measurements, contact sensors were used and placed in the profilometer construction to provide additional measurements and independent verification of thermogram readings. This study helped determine the time required for full thermal stabilization of the investigated contact profilometer as well as the temperature distribution of its construction. As a result, it was possible to correlate the temperature increase with thermal expansion and changes in the length of the X-axis measuring arm.

Figure 6 depicts the profilometer construction’s distribution of thermal fields at selected time intervals. Temperature measurement recording began when the device was turned on (thermally unstable device) and simultaneously from the initiation of the measuring tip movement. It should be noted that heating occurred in areas where drive motors and control electronics were located. The distribution of thermal fields across the entire device differs for the column and X-axis measuring arm drive. Additionally, valuable information from the recorded thermograms revealed that the X-axis drive’s increase in temperature was responsible for the movement of the measuring arm and the tip’s uniformity across the entire surface. Consequently, it was possible to correlate the temperature increase with the thermal expansion of its components and determine the synchronization error of individual paths/profiles, as shown in Figure 7.

Figure 7 illustrates the trend of average temperature over time for the profilometer drive, as measured by a thermal imaging camera in the marked area. Additionally, the value of the relative elongation of the X-axis arm (with respect to the Z-axis of the profilometer column) was highlighted using a laser interferometer. The elongation of the elements is proportional and closely related to temperature increases. Due to the small range of temperature changes not exceeding 3 °C, a linear dependence on thermal expansion was assumed, as expressed by Equation (1).

(1) $$l=l_{0}\cdot \left(1+\alpha \cdot ∆T\right),$$

where, l is the length of the material after change [m]; $l_{0}$ is the initial length [m]; α is the coefficient of linear expansion $\left[\frac{1}{K}\right];\;∆T$ is the temperature increase [K].

Figure 7 shows that full thermal stabilization occurred 12 h after the profilometer was turned on. The individual phases of heating are marked with colors. Three main intervals can be distinguished: 0–4 h (blue color) indicates the phase of heating the contact profilometer’s X-axis drive; 4–6 h (transitional phase—green colors) indicates the temperature stabilization phase; 6–12 h (red color) indicates the full temperature stabilization of the X-axis drive and the entire device. The measured temperature change for the X-axis arm drive from when the profilometer was turned on was 2.73 °C, and the relative elongation of the X-axis arm was 16.2 µm. It should be noted that this is the resultant value of expansion for each element forming the X-axis kinematic system.

**Figure 8:**
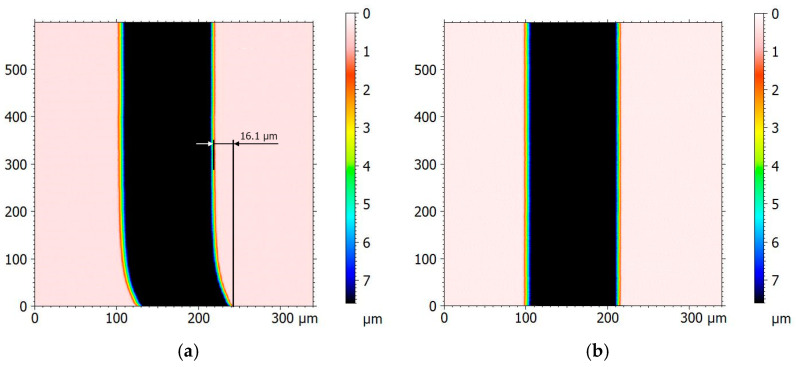
Representation of the reference scratch (fragment): (**a**) distorted scratch due to thermal effects and thermal expansion of the profilometer construction; (**b**) reconstructed scratch under stable environmental conditions.

Figure 8 presents an independent verification of the thermal expansion effect of the contact profilometer construction on the measured surface representation.

Figure 8 presents the reconstructed reference scratch (a fragment of the surface) measured while the profilometer was thermally unstable (Figure 8a). This illustration demonstrates the impact of improperly conducted measurements without thermal stabilization of the measuring device, leading to a lack of synchronization between paths/profiles. This finding is characterized by a nonlinear representation of the reconstructed scratch or surface. The shape of the distortion corresponds to a curve indicating a temperature increase. The distortion values of the scratch measured in MountainsMap correspond to interferometric measurements. The scratch measured under full stabilization of the contact profilometer (Figure 8b) shows no distortions and is characterized by a linear nature. The obtained results are consistent with the methodology described in Section 2.1 and confirm initial assumptions.

The methodology (Section 2.2) presented in Figure 5a precisely determines the effect of changes in the device’s center of gravity on profile measurements of surface irregularities. Each profile measurement (measurement cycle) can be divided into three characteristic phases:

– Starting the measurement in the resting position. This phase involves the movement of the X-axis arm (tip), which also initiates the displacement of the profilometer’s center of gravity;
– Linear movement of the X-axis arm (measurement of individual profiles);
– Return and approach the starting point. This movement, associated with returning to the starting point, is characterized by a slower arm movement speed in its final phase. This phase is required for the precise positioning of the measuring tip, which affects the synchronization of paths and the repeatability of the measurement.

**Figure 9:**
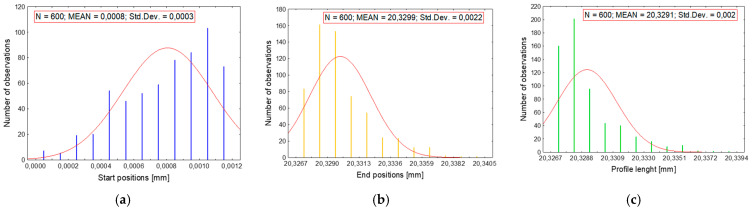
Method for determining the profilometer construction’s susceptibility: (**a**) MIN—positions reparability; (**b**) MAX—positions reparability; (**c**) MEAN—length profile reparability.

The statistical spreads of the measuring tip’s positioning for each state (START, END, repeatability of profile length) are shown in Figure 9. The repeatability of the measuring tip’s positioning at the start and end points, as well as the length of the measurement segment, is particularly important for the accurate synchronization of the paths and the representation of the spatial structure of the surface.

As seen in Figure 9, the uncertainty of the measuring tip’s position at the starting point (Figure 9a) does not exceed 0.3 µm. By contrast, the uncertainty value is 2 µm for each measurement segment (Figure 9c). The obtained information is particularly significant as it confirms the correctness of the adopted measurement methodology and the proper functioning of the device. At the same time, it indicates the priority positioning of the device’s measuring tip at the starting point from which path synchronization occurs.

**Figure 10:**
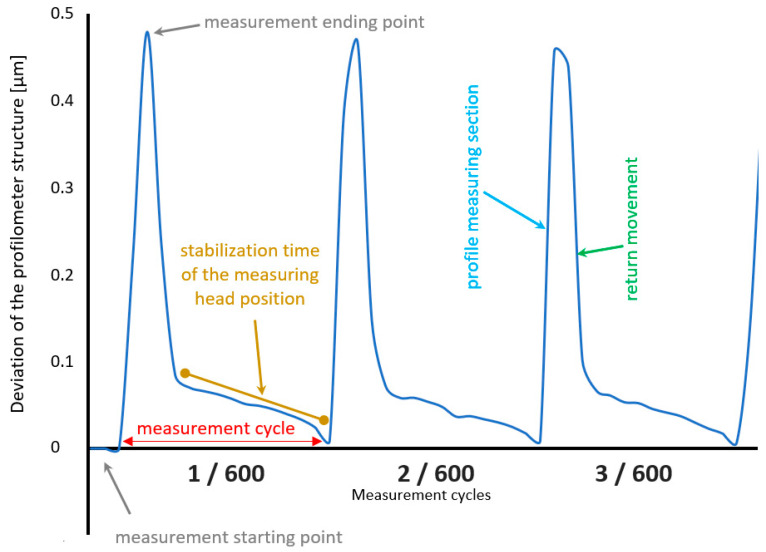
Interferometric measurement of the profilometer construction’s susceptibility due to the displacement of the center of gravity.

In Figure 10, a selected fragment of cycles 1–3 out of 600 measurement cycles is presented. The interferometer recorded the profilometer’s susceptibility (displacement/rotation in the Z-axis direction, according to the scheme in Figure 5a) to the measuring tip’s movement and changes in the center of gravity position of the X-axis drive assembly.

Each of the described phases of measurement head movement is reflected in the displacement plot recorded by the interferometer (Figure 10). This plot illustrates the relationship between the displacement of the center of gravity associated with the movement of the X-axis arm at each phase of the measurement cycle.

At the start of profile measurement (movement of the measurement head toward the Z column), there is an increase in distance between the reference and measurement mirrors in the optical path of the laser interferometer. This difference is visible in the plot as a rising slope. The measurement head’s return movement causes the center of gravity to move in the opposite direction relative to the device column, decreasing the distance between the mirrors (descending slope). The change in the speed of the measurement head during the return phase ensures accurate positioning relative to the starting point, which is also visible on the displacement plot as a shorter segment with a smaller slope.

The registered displacement value represents the overall susceptibility of the entire profilometer construction. It also represents deviations in the profilometer’s column and rotation at the mounting point of the X-axis drive assembly. The displacements observed in the plot are determined at a specific interferometer mirror position. This mirror was located at a known distance from the vertical Z-axis and the rotation axis of the X-axis drive of the profilometer. Knowing the distance from the theoretical intersection point of the profilometer axes (X, Z), it is possible to calculate the vertical displacement value at any distance from the measurement head extension. Based on the experimentally determined displacement values and calculations, leveling deviations not exceeding 1 µm over the measured section were determined.

**Figure 11:**
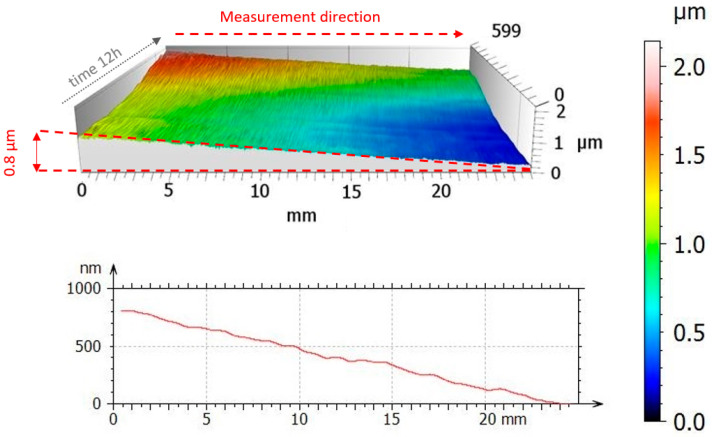
Surface inclination due to the profilometer construction’s susceptibility along with a representation of the cross-sectional profile.

Interferometer-based calculations and measurements were verified by analyzing the average waviness profile and the reconstructed surface that underwent no processing, including leveling and filtering (Figure 11).

Figure 11 presents raw data from measurements of the reconstructed surface with measurements of the same profile over time. Although precise leveling was ensured based on relevant information from TurboWAVE, which controlled the profilometer, Figure 11 shows a deviation from the level of the reconstructed surface at 0.8 µm. The deviation value is close to the calculated value. This relationship confirms interferometric measurements, confirming the influence of arm movement at the measuring tip and the displacement of the center of gravity on the profilometer’s construction.

The applied method has certain limitations. It requires stable environmental conditions for the optical interferometer system, limiting the device’s temperature drift, and precise alignment with the parallelism of outgoing and reflected beams. A major limitation of the method is ensuring an uninterrupted laser beam, which restricts its ability to measure displacements in a single-axis direction (in the case of a single-channel interferometer). It is worth noting that using an optical interferometer system must be mounted to the device, which can be challenging in some cases, or may require special mounts dedicated to specific applications. Despite the aforementioned limitations, this method is highly effective and demonstrates significant applicability in the discussed study.

## 4. Conclusions

This article presents selected errors in tactile profilometry affecting the representation of surface irregularities. After analyzing the research results, the following conclusions were formulated from two separate aspects:

– The influence of internal heat sources on the synchronization of paths and repeatability of measuring tip positioning in the direction of the X-axis for the studied tactile profilometer was determined;
– The effects of thermal expansion of the profilometer’s X-axis drive on the spatial representation of the surface were determined. For this thermally unstable device, the value was 16.1 µm and characterized by an asymptotic trend;
– The time required for the thermal stabilization of the profilometer’s X-axis drive was between 6 and 12 h from when the device was turned on;
– The repeatability of measuring tip positioning in this case is best for the starting point. The positioning uncertainty did not exceed 0.3 µm, thereby influencing the accurate synchronization of paths in spatial surface roughness measurements;
– The tactile profilometer construction’s susceptibility was due to changes in the center of gravity caused by movement in the X-axis direction of the measuring tip during profile/surface measurement;
– Despite leveling the measuring head, the reconstructed surface deviated from the level of 0.8 µm in a 25 mm section within the selected range of motion.

Understanding these relationships will help reduce errors related to measurement procedures and equipment preparation for research. It will also allow a proper assessment of surface geometry.

Based on the conducted research and formulated conclusions, we intend to expand these studies according to the adopted methodology for other devices, particularly optical profilometers based on the focus variation technique. In this technique, the objective lens moves along the Z-axis to register data for individual cross-sections. Thermal conditions and their impact on the behavior of the optical profilometer’s structure and the optical system’s positioning capabilities for adopted sampling resolutions will also be considered. 

## Data Availability

Data is contained within the article.
